# Are we there yet? The long walk towards the development of efficient symbiotic associations between nitrogen-fixing bacteria and non-leguminous crops

**DOI:** 10.1186/s12915-019-0710-0

**Published:** 2019-12-03

**Authors:** Vânia C. S. Pankievicz, Thomas B. Irving, Lucas G. S. Maia, Jean-Michel Ané

**Affiliations:** 1 Department of Agronomy, University of Wisconsin, Madison, WI USA; 2 Department of Bacteriology, University of Wisconsin, Madison, WI USA

## Abstract

Nitrogen is an essential element of life, and nitrogen availability often limits crop yields. Since the Green Revolution, massive amounts of synthetic nitrogen fertilizers have been produced from atmospheric nitrogen and natural gas, threatening the sustainability of global food production and degrading the environment. There is a need for alternative means of bringing nitrogen to crops, and taking greater advantage of biological nitrogen fixation seems a logical option. Legumes are used in most cropping systems around the world because of the nitrogen-fixing symbiosis with rhizobia. However, the world's three major cereal crops—rice, wheat, and maize—do not associate with rhizobia. In this review, we will survey how genetic approaches in rhizobia and their legume hosts allowed tremendous progress in understanding the molecular mechanisms controlling root nodule symbioses, and how this knowledge paves the way for engineering such associations in non-legume crops. We will also discuss challenges in bringing these systems into the field and how they can be surmounted by interdisciplinary collaborations between synthetic biologists, microbiologists, plant biologists, breeders, agronomists, and policymakers.

## Introduction

Nitrogen is an essential component of life, required for building proteins and DNA, and despite being abundant in the atmosphere, only limited reserves of soil inorganic nitrogen are accessible to plants, primarily in the form of nitrate and ammonium. Thus, agricultural yields are often limited by nitrogen availability [1]. This limitation was battled for centuries by crop rotation or co-culture with legumes and the use of fertilizers in the form of animal waste, wood ash, or seaweed. At the beginning of the 20^th^ century, two German chemists, Fritz Haber and Carl Bosch, invented a process allowing nitrogen fixation, the conversion of dinitrogen into ammonium, on an industrial scale [1, 2]. The use of synthetic fertilizers was the main factor for drastically increase crop production during the Green Revolution, especially in developing countries, and the subsistence of nearly half of the world population is currently dependent on the use of such fertilizers [3]. Breaking the triple bonds of dinitrogen requires vast amounts of energy (1–2% of the global energy supply) and about one ton of natural gas is dedicated to the production of one ton of synthetic nitrogen fertilizers [4, 5]. Not surprisingly, the cost of fertilizers is highly dependent on the price of natural gas, which is currently low due to the practice of hydraulic fracturing or fracking [6]. However, the dependence of so much food production on natural gas, a finite resource, is concerning. Ironically, even biofuel production (e.g., corn ethanol) depends on the use of synthetic fertilizers and therefore fossil fuel, which defeats the very purpose of biofuels. All these examples reveal that nitrogen availability for crops is a threat to the sustainability of our agricultural systems, economy, and food supply.

Besides these global sustainability considerations, the intensive use of fertilizers also creates specific issues in developed and developing countries. Addition of Haber-Bosch derived nitrogen, sometimes more than 200 kg N ha^−1^yr^−1^, has increased yields but also led to the contamination of groundwater and eutrophication of rivers, causing massive community shifts for inland and coastal aquatic microbiota and impacting human health [7–9]. In contrast, subsistence farmers are unable to access fertilizers at an affordable price. Lack of local production and poor transportation infrastructure also contributes to low yields and, thus, cycles of food insecurity and poverty [10].

Bacteria and *Archaea* have been fixing atmospheric nitrogen for hundreds of millions of years [11]. This biological nitrogen fixation accounts for much of the nitrogen input of natural systems, considerably more so than rock weathering or lightning [12]. Biological fixation in prokaryotes is performed by the nitrogenase complex, a metalloenzyme complex composed of the catalytic protein dinitrogenase, and an ATP-dependent electron-donating iron protein, the dinitrogenase reductase. The catalytic domain of dinitrogenases commonly contains a molybdenum-iron cofactor, but some species use two other classes of dinitrogenases, defined by the presence of vanadium-iron or iron-only cofactors [13]. The nitrogen fixation genes (commonly referred to as *nif* genes) encode the components of nitrogenase and other regulatory proteins. The *nifHDK* operon encodes the dinitrogenase and the dinitrogenase reductase, but additional proteins are required to produce a fully functional holoenzyme. About 20 *nif* genes have been found in nature across the three classes of nitrogenases [13–15].

**Fig. 1:**
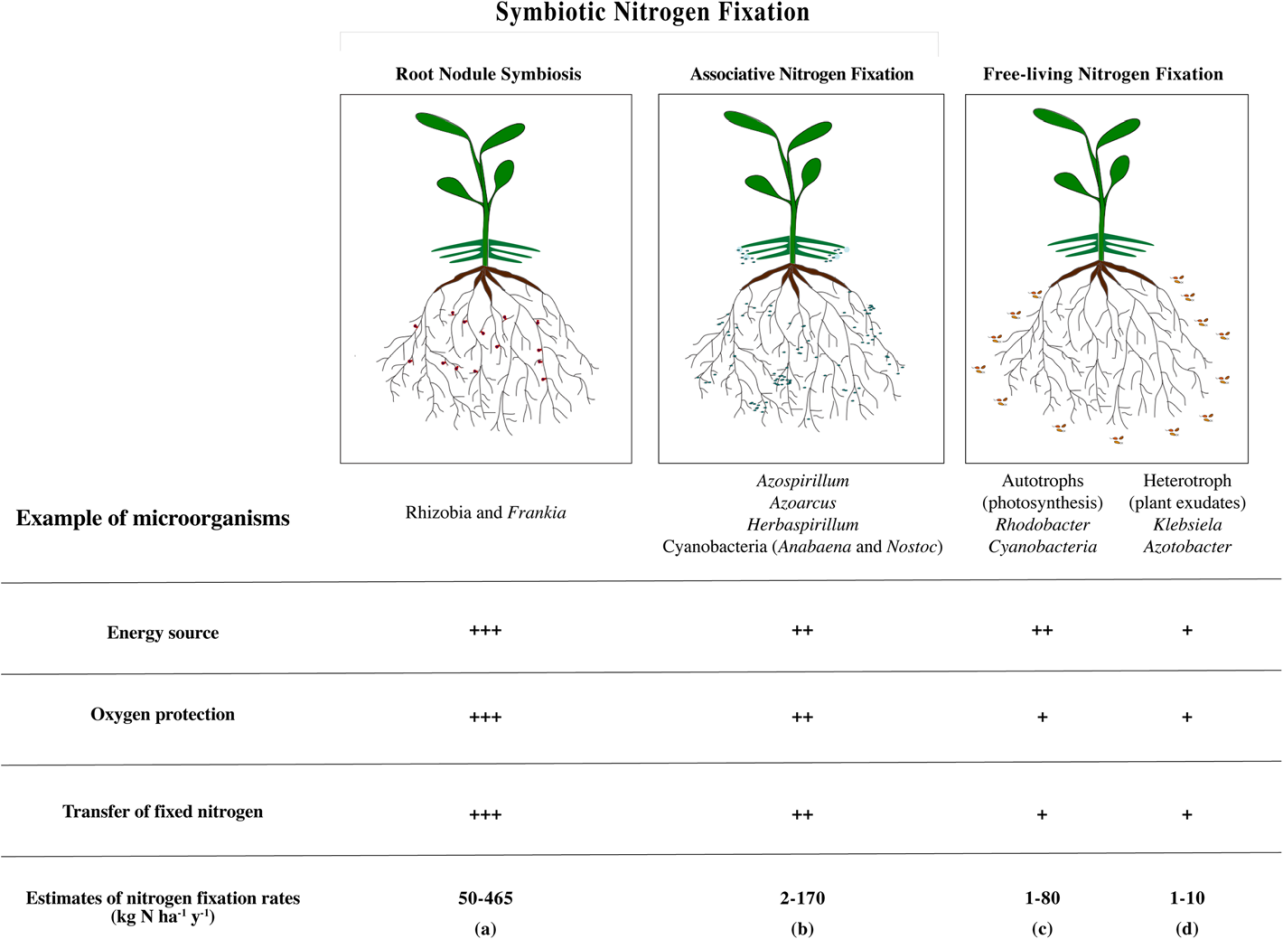
Different types of nitrogen-fixing associations with plants. The three challenges of biological nitrogen fixation are solved with different efficiency by these types of interactions—energy source, oxygen protection, and transfer of fixed nitrogen to the plant. The efficiency of each bacterial partner is indicated by + (low), ++ (moderate), or +++ (high). The nitrogen fixation rates depend on the efficiency of the interaction. **a** Root nodule symbiosis, 50–465 kg N ha^−1^ y^−1^ [17, 18]; **b** associative nitrogen fixation, 2–170 kg N ha^−1^ y^−1^ [19–23]; and **c, d** free-living nitrogen fixation, 1–80 kg N ha^−1^ y^−1^ [24–26]

Nitrogen-fixing prokaryotes, also called diazotrophs, can be free-living or exist in symbiotic associations with Eukaryotes, with examples including fungi (*Geosiphon*), sponges (*Dysidea*), termites, and plants [16]. A successful symbiosis requires an appropriate host and diazotrophic partner, combined with environmental conditions to allow nitrogen fixation. Diazotrophic bacteria are highly diverse and are found in various ecological niches (free-living or in association with different organisms; Fig. 1) and have a wide range of metabolic characteristics [27–29]. In plant–bacteria interactions, the energy-intensive nitrogen fixation is powered by photosynthates from the plant, in exchange for a portion of the fixed nitrogen. Most of the time, "symbiotic nitrogen fixation" has referred only to symbioses leading to the development of root nodules. By definition, however, symbiosis is a long-term association between two different organisms that is beneficial for at least one of them [30]. Associative nitrogen fixation obviously meets this definition, as the plant benefits from growth promotion (both via increased nitrogen nutrition and several other benefits) and the bacteria gains carbon from plant photosynthesis. Thus, in this review, we will refer to both root nodule symbioses and associative nitrogen fixation as "symbiotic nitrogen fixation" (Fig. 1). Some publications already employ these terms similarly [31], but we believe that the community should also adopt this terminology more widely.

### The root nodule symbiosis and the unexploited diversity of nitrogen-fixing microorganisms in nature

Root nodule symbioses are only found in plants of a monophyletic clade often referred to as “FaFaCuRo” (*Fabales*, *Fagales*, *Curcubitales*, and *Rosales*) but are incredibly diverse in modes of infection by rhizobia or *Frankia*, nodule anatomy, and metabolism [32–35]. Associations between legumes and rhizobia are so efficient that legumes are found in a wide range of environments across the globe and used in nearly all cropping systems [36]. Genetic approaches have been essential to the dissection of the molecular mechanisms that control the establishment of these associations [37–39]. Genetic tools were first developed in rhizobia, with rhizobial mutants unable to trigger the development of root nodules, allowing the identification of *nod*, *nol*, and *noe* genes [40–42]. Some *nod* genes encode regulatory NodD proteins that bind to diffusible signals present in legume root exudates (flavonoids, isoflavonoids, and betaines) and regulate the expression of other *nod* genes that control the production of Nod factors [15]. Nod factors are lipo-chitooligosaccharides (LCOs) with a short chitin backbone of three to five residues of N-acetylglucosamine, with an acyl chain at the reducing end [43]. Nod factors are decorated with various substitutions (methyl, acetyl, fucose, arabinose, and others) that are the primary determinant of the often high levels of host specificity observed in the rhizobia–legume symbiosis [44]. Symbiotic interaction between the actinobacteria *Frankia* and actinorhizal plants may use different recognition factors, in which as yet unknown diffusible signals drive the pre-infection responses, instead of chitin-based signals used by rhizobia [45]. Bacterial exopolysaccharides are also often required and recognized by specific receptors for successful colonization [39, 42]. The “common *nod* genes” are found in most rhizobia and control the production of the lipo-chitooligosaccharide backbone. In contrast, "specificity *nod* genes" are present in some but not all rhizobial strains and control the addition of substitutions on this chitin backbone and therefore host specificity [46]. For instance, a *Sinorhizobium meliloti nodH* mutant is no longer able to nodulate its natural host alfalfa but nodulates vetch [47]. A few bradyrhizobia have been shown to nodulate some legumes in the absence of *nod* genes, but the vast majority of rhizobia require *nod* genes and Nod factors to associate with their legume hosts [48–50].

The genetic mechanisms that control root nodulation have been deeply dissected in two model legumes, *Medicago truncatula* and *Lotus japonicus* [51–55]. The host mechanisms include three distinct processes that can be uncoupled genetically: mutual recognition, colonization (often called infection), and nodule development (organogenesis) [56, 57]. Mutual recognition begins with the perception of Nod factors by lysin motif receptor-like kinases [58, 59]. A mechano-stimulation from the microbes may modulate symbiotic signaling [60]. The activation of these receptors leads to the activation of the “common symbiosis pathway” (CSP), a signaling pathway controlling intracellular colonization and presumably adapted from the more ancient symbiosis between land plants and arbuscular mycorrhizal fungi [61–63]. In legumes, the CSP induces expression of transcription factors including *NODULE INCEPTION* (NIN) and members of the NF-Y family, which control nodule organogenesis in concert with the cytokinin signaling pathway [53, 56, 64, 65]. Strikingly, NIN-like and NF-Y proteins are also involved in lateral root organogenesis in many plants, although cytokinin often acts to repress lateral root initiation [66–68].

Root nodules and lateral roots are both lateral root organs. Their similarities and differences have been the subject of debates over decades. The nodules of some legume species do not have a persistent meristem (determinate), but the nodules of many legumes have a persistent meristem (indeterminate) like lateral roots [69]. Classically legume root nodules have been differentiated from lateral roots by the presence of peripheral vasculature, whereas lateral roots have central vasculature. However, some actinorhizal plants have nodules with a central vasculature [34]. Genetic evidence supports the idea that the mechanisms used by plants for developing nodules have been co-opted and slightly modified from those used to form lateral roots in most plants [70]. For example, legume mutants in the transcription factor *NOOT* form nodules with a meristem that reverts to a lateral root identity [71]. Altogether, genetic and evolutionary studies indicate that root nodulation evolved from recruiting pre-existing mechanisms of arbuscular mycorrhizal associations and lateral root development, connecting NIN and possibly other proteins into the CSP, and bringing auxin and cytokinin together to drive nodule development [72–75].

The root nodules of the legume plants provide an excellent environment for nitrogen fixation, with rates of 50–465 kg N ha^−1^ yr^−1^ in agricultural settings, and has been a significant focus of the agronomic community over the last decades [17, 18, 76]. Intracellular nitrogen-fixing symbioses outside the legume and actinorhizal lineages are rare. *Gunnera* species host the cyanobacteria *Nostoc* in stem glands, and this symbiosis can also provide substantial amounts of fixed nitrogen (15 kg N ha^−1^ yr^−1^) [77]. Associations between plants and epiphytic or free-living diazotrophs can also provide significant amounts of nitrogen to the host plant. These associative symbioses are quite diverse. For instance, the interactions between lichens and mosses and cyanobacteria can contribute up to 3 kg N ha^−1^ yr^−1^ to subarctic and boreal forest communities [19, 78]. Rice paddies are naturally fertilized by "green manure", comprised of aquatic ferns (*Azolla*) extracellularly associated with *Anabaena azollae* [1, 79]. Many other plants accommodate *Nostoc* extracellularly, including cycads on modified (collaroid) roots and in slime-filled cavities in many bryophytes [80]. The nitrogen amounts fixed by these symbioses are poorly evaluated [19]. Endophytic diazotrophs, such as *Gluconacetobacter diazotrophicus*, *Herbaspirillum seropedicae*, *Herbaspirillum rubrisubalbicans*, and *Burkholderia silvatlantica*, can fix nitrogen in the vasculature and intercellular spaces of sugarcane stems [81, 82]. Diazotrophs, including *Herbaspirillum* species, living in mucilage released from the aerial roots of maize landraces from Sierra Mixe, Mexico, can provide up to 82% of the host nitrogen [83].

Plants also benefit from nitrogen fixed by bacteria in the soil, which obtain their energy either from degrading organic matter in the ground (heterotrophs) or from photosynthesis (autotrophs), but the contribution of this fixed nitrogen to crops is lower than from symbioses [24, 84]. In 2016, Ladha et al. estimated that biological nitrogen fixation in the rhizosphere of rice, wheat, and maize contributed up to 25% (13–22 kg N ha^−1^ yr^−1^) of the total nitrogen in harvested grain, but it was not possible to quantify the respective contributions of associative and free-living fixation [20].

Symbiotic nitrogen fixation contributes to the growth-promotion effect seen in plant growth-promoting rhizobacteria, although it is generally not the only benefit that these bacterial symbionts provide to the plant host. Rhizobacteria can increase plant access to other nutrients, enhance defense against pathogens or abiotic stresses, and secrete plant hormones [85–88]. It is often challenging to differentiate the contribution of biological nitrogen fixation from plant growth promotion by other factors [89–91]. The techniques used to evaluate how much nitrogen is fixed and transferred to the plants have strengths and pitfalls (described in Table 1). These issues have led to many conflicting reports and confusion in the literature. We believe that proper estimation of nitrogen fixation can only come from using several if not all of the techniques mentioned in Table 1 [83].

**Table 1:** Estimating the contribution of biological nitrogen fixation

Determining the rate of nitrogen fixation is a difficult task, especially in field conditions. Five categories of techniques have been used, and all of them have their pitfalls.	
(1) The acetylene reduction assay (ARA) is a sensitive and accurate method of assessing nitrogenase activity, via the indirect measure of reduction from acetylene to ethylene by nitrogenase. However, different types of nitrogenases reduce acetylene differently, leading to discrepancies with other methods, and this method is challenging in field conditions due to the flammable acetylene gas and difficulties in tightly enclosing the plant. Most importantly, this technique cannot evaluate how much of the fixed nitrogen is assimilated by the plant.	
(2) The ^15^N natural abundance technique relies on the higher abundance of this naturally occurring and stable nitrogen isotope in most soils [92]. A diazotroph acquiring its nitrogen from the air and its host will, therefore, have a lower ^15^N abundance than plants only obtaining their nitrogen from the soil. Variations in isotope ratios are reported as ∂-values, commonly expressed in parts *per mil* (‰). These variations are measured using isotope-ratio mass spectrometry. This technique is high throughput and can be performed in fields. The stable nature of ^15^N isotopes allows storing and shipping samples efficiently. Unfortunately, variations in ^15^N abundance across the experimental field or from a geographical location to another and soil horizons can lead to artifacts, and the use of abundant controls, including soil samples, is required.	
(3) ^15^N isotope dilution is a variant of the previous technique where the soil is enriched with a ^15^N-enriched nitrogen source to increase the differential between the ground and the air and limits the natural variations in ^15^N abundance. However, the cost of ^15^N-enriched nitrogen restricts the scale of these experiments. ^15^N-enriched sources can also move vertically or horizontally during the growing season, which mandates frequent soil sampling for controls [92, 93].	
(4) Another ^15^N-based technique, called ^15^N gas enrichment, is conceptually the reverse of the previous ones. In this case, dinitrogen from the air is labeled with ^15^N and the incorporation of ^15^N in bacteria and its host plant indicates that they acquired some of their nitrogen from the air. This technique is one of the best pieces of evidence to prove that plants obtained nitrogen through nitrogen fixation. However, bacterial contaminations must always be considered as another source of N reaching the host. Sensitivity can be enhanced using radioactive nitrogen isotopes, such as ^13^N, but these are challenging to use given their short half-life [94]. Determining if ^15^N was incorporated in the host tissues is best achieved by mass spectrometry imaging or by extracting plant-specific metabolites such as chlorophyll [95, 96].	
(5) Nitrogen-balance experiments evaluate the amount of nitrogen acquired by the plant from the soil and the total amount of nitrogen in the plant. The difference between the two measurements gives the amount of nitrogen from the air. However, evaluating soil nitrogen is difficult, introducing a significant level of uncertainty in these evaluations.	

### Biological challenges for efficient nitrogen-fixing symbioses

Extending efficient symbiotic nitrogen fixation from legumes to cereals has been a dream of agronomists since the understanding of the benefits behind the legume crop rotation system [97]. As early as 1917, scientists attempted to cultivate the rhizobia from legumes and inoculate these into other species [98]. To date, however, none of these attempts to transfer the complex root nodule to non-legume plants has succeeded. Symbiotic nitrogen fixation can take many forms in nature, but the main challenges solved by these different biological systems are quite similar: energy source, oxygen protection, and efficiency of nutrient exchange. The same problems also face any new approach that aims at improving or creating a nitrogen-fixing symbiosis.

1. Nitrogen fixation is energy expensive, with the reduction of dinitrogen into ammonia requiring at least 16 ATP per dinitrogen fixed (Table 2). However, the real cost is estimated to be 20–30 ATP, accounting for the production of the nitrogenase complex, the reductive power, and recycling the toxic dihydrogen waste resulting from the process [99, 101].
2. The catalytic [4Fe-4S] cluster of dinitrogenase, which is exposed between the subunits, is permanently oxidized in minutes, while the dinitrogenase reductase—the ATP-dependent iron protein—is inactivated in seconds [102–104]. Thus, the entire complex is highly vulnerable to destruction by molecular oxygen. This oxygen sensitivity leads to the oxygen paradox of biological nitrogen fixation, as the most efficient source to produce ATP is aerobic respiration, which requires the presence of oxygen [105]. One solution to this paradox is to avoid oxygen entirely, respiring on sulfate, hydrogen, or metal ions. These systems are not possible in conditions in which plants can grow, so active diazotrophs must tightly regulate internal oxygen tension to supply aerobic respiration while limiting harm to nitrogenase. In legume nodules, the physical structure, including the suberin in the endodermis, acts as a physical barrier to oxygen diffusion and the leghemoglobin acts as an oxygen buffer to maintain a low oxygen tension [106]. To preserve respiratory capacity and energy production, the terminal oxidase of the electron transport chain of rhizobia binds oxygen more strongly than in most microbes, even pulling oxygen out of the leghemoglobin [107]. In non-legume symbioses, the viscous mucus excreted by maize aerial roots limits oxygen diffusion while the root and microbes in it consume oxygen, leading to low internal oxygen tension [83, 108]. Bacteria produce exopolysaccharides and biofilms on root surfaces to similar effect [109, 110]. Autotrophic cyanobacteria must produce oxygen from photosynthesis to power fixation, protecting their nitrogenase either by separating the nitrogenase physically in dedicated heterocyst cells or temporally by fixing nitrogen only at night. Soil diazotrophs like *Azotobacter* contain an additional respiratory chain dedicated to consuming oxygen to maintain an anoxic cytoplasm [111]. This is complemented by conformational protection, where iron-sulfur Shethna proteins form part of the nitrogenase complex and cover the active site in the presence of oxygen, temporally inactivating the enzyme but preventing permanent oxidative damage [111–113].
3. The efficiency of nutrient exchange between the two partners is also critical. Fixed carbon must be fed to the symbiont for energy and nitrogen exported to the host while limiting losses to other organisms or the environment. In root nodules the bacteria fix nitrogen within plant cells (endosymbiosis), which provides a large surface of contact to exchange nutrients between host and symbionts with striking structural and molecular similarities to mycorrhizal arbuscules [114–116]. In aerial roots of Sierra Mixe maize, nitrogen released by the bacteria in the gel is actively taken up by aerial roots.

**Table 2:** Idealized nitrogen fixation equation

N_2_ + 8 H^+^ + 8 e^-^ + 16 ATP ➔ 2 NH_3_ + H_2_ + 16 ADP + 16 Pi [99, 100]	

Energy must be expended to support bacterial growth even in the most efficient legume systems, increasing the cost of nitrogen fixation for the plant. Estimating this cost is complicated, given that additional nitrogen leads to more photosynthesis, but a loss of 5.6–8.0 g of carbon per gram of reduced nitrogen obtained by legumes appears a reasonable estimate. This represents around 30–40% efficiency relative to the theoretical cost of 2.5 g of carbon per gram of reduced nitrogen [117]. One solution to this inefficiency loss would be to express the nitrogenase complex directly in the plant. This would also prevent losses during the nutrient exchange but is a much more complex technical challenge.

#### Manipulating the bacterial partner to increase biological nitrogen fixation in non-leguminous plants

The search for microbes to improve both monocot crop nitrogen nutrition and development is a long-standing aspiration [118–120]. After the ‘70s, with the efforts of Dr. Johanna Döbereiner, the association between diazotrophs and cereal crops received more attention. *Azotobacter* and *Beijerinckia* were first isolated from sugarcane and cereal grasses in 1961 [121]. *Enterobacter cloacae* was found in corn roots in 1972, and in rice, wheat, and tropical grasses in 1973 [122]. *Spirillum* sp. strains were first isolated in 1975 from surface sterilized maize roots, and their nitrogenase activity demonstrated [123]. In the ‘80s, the endophyte *Herbaspirillum seropedicae* was isolated from maize, sorghum, and rice, and *Gluconacetobacter diazotrophicus* from sugarcane [124, 125]. After the advent of the acetylene reduction assay (ARA), it was possible to test bacteria for nitrogen-fixation ability directly [126]. Diazotrophs isolated from sugarcane and cereals, including but not limited to *G. diazotrophicus*, *Herbaspirillum frisingense*, *H. seropedicae*, and *Azospirillum brasilense*, were shown to contribute at various levels to the plant nitrogen requirements via nitrogen fixation under laboratory and field conditions [21, 22, 127–131]. Next-generation sequencing made possible the identification of free-living, endophytic, and epiphytic diazotrophs on a massive scale, using genes encoding core proteins of the nitrogenase complex as markers for screening metagenomes [132–134]. However, the presence of these genes merely reflects the potential of the microbiota for nitrogen fixation [135–137]. We believe that these DNA-based surveys should be more systematically complemented with transcriptomic and possibly proteomic approaches to determine if these *nif* genes are actually expressed. Global methods are also not sufficient to evaluate the benefits provided to the host, which requires isolation. The right nitrogen-fixing symbiont for crops must both be an efficient colonizer of the root system and release a significant portion of its fixed nitrogen to the plant. Ideally, it would keep fixing nitrogen even in fertilized fields.

Attempts to isolate better ammonium releasers have used ethylenediamine to deregulate glutamine synthase. One example, *Azospirillum brasilense* HM053, allowed the model C4 monocot *Setaria viridis* to grow in nitrogen-free media and promoted wheat growth in laboratory conditions [129, 131, 138] and maize growth in field conditions [139]. This effect appears common, as ethylenediamine-treated *Pseudomonas* sp. also increased the biomass of plants grown under nitrogen-limiting conditions [140].

Significant progress has been made in understanding the biochemical, physiological, and ecological aspects of diazotroph associations with cereals. Many diazotrophs also promote plant growth through other mechanisms, such as the production of plant hormones, phosphate solubilization, and the acquisition of other nutrients like calcium, potassium, iron, copper, magnesium, and zinc (reviewed in [141, 142]). These mechanisms can further increase plant nitrogen access by increasing root growth and relieving nutrient deficiencies. However, the genetic mechanisms that drive the establishment of cereal–microbe interaction are still poorly understood, and this must be corrected if we are to exploit these associations more effectively. Genetic tools have been developed to study the endophytic diazotroph *Azoarcus* sp. BH72, and allowed the characterization of the molecular mechanisms controlling its interaction with plants [143]. Interestingly, *Azoarcus* sp. BH72 induced to fix nitrogen cannot be returned to culture, suggesting that it undergoes terminal differentiation in a way perhaps similar to the differentiation of rhizobia into bacteroids in the *rhizobium*–legume symbiosis [144]. Recently, Faoro et al. [145] isolated a new strain, *Azoarcus olearius* DQS-4T, in oil-polluted soils. This DQS-4T strain demonstrated significant plant growth promotion activity and an active nitrogenase [145]. This finding highlights the importance of continuing to prospect, in a wide range of environments, for better nitrogen fixers, better colonizers, and plant growth promoters.

### Genetic engineering strategies towards better nitrogen-fixing microsymbionts

Engineering microsymbionts may make it possible to confer nitrogen-fixing ability on non-diazotrophs or to improve the benefits of natural associations between diazotrophs and crops significantly [146]. The transfer of fixation capacity to a non-diazotroph was first achieved in 1971, with the transfer of a *nif* cluster from *Klebsiella pneumonia*e into *Escherichia coli* [147]. Subsequently, many researchers have produced transgenic bacteria capable of fixing nitrogen, discovering the minimum set of *nif* genes required for the production of a functional nitrogenase [148–150]. An exciting goal of engineering increased nitrogen fixation is to remove the inhibition of nitrogenase by nitrogen and oxygen and to alter metabolism so that more ammonium is released to the plant rather than incorporated into bacterial metabolism. A *nifL* mutant in *Azotobacter vinelandii* was isolated in the 90s that could fix and release nitrogen even in the presence of 15 mM ammonium [151]. Deletions of *nifL* in *Azotobacter* and *Pseudomonas* also improved the excretion of ammonium and increased expression of the *nif* genes in the presence of oxygen [152, 153]. Manipulating the bacterial ammonium assimilation pathway is also a straightforward strategy to increase the amount of ammonium released by diazotrophs. Mus et al. achieved that by mutation of *glnE* in *A. vinelandii*, preventing the posttranslational repression of glutamine synthetase by ammonium [154]. This improved diazotrophic growth but impaired growth and reduced fitness on ammonium-containing medium. Similarly, deleting the ammonium transporter *amtB* led to increased ammonium excretion [153]. Also, decreased glutamine synthetase activity resulted in ammonium release in *A. vinelandii glnA* mutants and *A. caulinodans glnB* or *glnK* mutants [155, 156]. For a more significant review of nitrogen-fixation regulation see [13, 146].

As mentioned previously, a significant challenge of biological nitrogen fixation is that the nitrogenase is irreversibly inactivated by oxygen [113, 157]. It had been reported that *Streptomyces thermoautotrophicus* UBT1 possesses a novel class of nitrogenase that was supposedly insensitive to oxygen. This would have been a significant finding. Unfortunately, further studies demonstrated that the described nitrogenase is not present in the *S. thermoautotrophicus* genome, and the diazotrophic phenotype could not even be recapitulated [158, 159]. It is thus unclear if an oxygen-insensitive nitrogenase is even possible. However, efforts are in progress to transfer oxygen protection systems, like the Shethna protein of *A. vinelandii*, to other diazotrophs [160].

### Efficient biological nitrogen fixation requires close interactions between bacteria and the plant host

Plant growth promotion is the result of interactions between soil type, microbiota, and the host plant. Benefits to the plant can come from a wide array of mechanisms [161, 162]. Unfortunately, much of the work on these benefits has been limited to describing phenotypes rather than the underlying genetics. The host genotype is also an essential player in defining the microbial communities and their benefits to the interaction partners [163]. *Azoarcus* is known to be a very efficient colonizer and was the first non-rhizobial diazotroph with a sequenced genome [143]. Using mutagenesis studies and labeled bacteria, the mechanisms involved in the *Azoarcus*–rice interaction have been well described, but not yet translated into practical applications to the field. Early field studies for *Azospirillum* seem more promising [164–167]. *Azospirillum* is part of a broad group of plant growth-promoting bacteria, together with endophytic diazotrophs from the genera *Herbaspirillum*, *Gluconacetobacter*, *Klebsiella*, and *Burkholderia* [168–171]. Infection and colonization of grasses by these endophytes have been well described at the microscopic and physiological levels.

In the genus *Pseudomonas*, several species can colonize plants and promote plant growth efficiently. The transfer of the nitrogenase from *Pseudomonas stutzeri* to the non-nitrogen fixing root-associated *Pseudomonas protegens* Pf-5 was suggested to supply nitrogen to several crops [128, 130], but, to our knowledge, these results have yet to be replicated by other teams. The same authors showed that heterologous polyhydroxybutyrate production might regulate nitrogenase activity. Polyhydroxybutyrate is a carbon storage polymer that can be mobilized under stressful physiological conditions, increasing the survival of bacteria in the soil. Indeed, recently, *H. seropedicae* strains overproducing polyhydroxybutyrate were shown to have better colonization fitness when compared to wild-type strains [172]. This highlights the importance of studies that integrate nitrogen fixation into bacteria with better plant colonization ability. Finding genes that improve this colonization ability will open new avenues to increase inoculant efficiency and survival between crops.

Another approach towards searching for better colonizers are microbiome studies and, in particular, those going beyond 16S-based classification to look at functional genes. Such efforts include the Earth Microbiome Project, which collected information for more than 30,000 microbiota samples across the globe [173]. Recent work compared 3837 bacterial genomes, aiming to identify plant-associated gene clusters, and found that plant-associated bacteria genomes encoded more carbohydrate metabolism genes than related non-plant-associated genomes and determined 64 plant-associated protein domains that possibly mimic plant domains [174]. This can potentially lead us to a comprehensive set of genes that directly affect the symbiotic interaction between bacteria and non-legume hosts.

#### The search for better plant hosts for nitrogen-fixing bacteria

In the quest for nitrogen-fixing crops, a lot of the community efforts have been focused on legumes, which, as reviewed previously, led to a wealth of knowledge on root nodule symbiosis, but the practical applications of this knowledge are probably a long-term goal. Previous attempts involved the transfer of seven core CSP genes from *M. truncatula* to a variety of non-fixing eudicots and were unsuccessful at inducing nodulation [175]. We now know that these species already contained functional orthologs of these genes, as they are conserved for signaling in the ancestral arbuscular mycorrhizal symbiosis. A complete rebuild of the nodulation pathway in a non-host would probably require a large number of genes and may as yet be impossible with our current understanding of the symbiosis. But, efforts to ‘brute force’ a new host by transfer of all or a substantial core set of nodulin genes to monocotyledonous crops is likely unnecessary. The very concept of ‘nodulin’ genes is questionable as large-scale transcriptomic approaches demonstrate that many of these genes are expressed in other tissues or conditions [176]. Most if not all genes involved in nodulation have been repurposed from existing conserved families, including roles in homeotic flower development (NOOT) [177], root architecture in response to nitrogen (NIN family transcription factors) [66], and the autoregulation of nodulation pathway [178] and defense (Nodule cysteine-rich peptides) [35, 179–181].

A more efficient approach is probably to exploit the conservation of most ‘nodulin’ genes outside of the FaFaCuRo clade, taking an evolution-guided ‘minimal change’ approach to engineering. Griesmann et al. suggested that the change that enabled nodulation was the coordination of expression of ‘nodulin’ genes, rather than the appearance of new genes not seen outside the FaFaCuRo clade [35]. The same idea, that the evolution of nodulation was a gain of regulatory elements rather than protein coding sequences, was also proposed by Doyle [182]. Taking this evolution-guided approach step by step through the stages of the nodule symbiosis, we first observe that all plants release at least the basal flavonoid naringenin, which is known to activate *nod* gene expression in several rhizobial species [183]. Thus, it is likely easier to move the *NodD* gene from these species to other rhizobia than alter flavonoid metabolism in the plant. All mycorrhizal plants contain LCO receptors capable to some extent of binding rhizobial Nod factors, although these ‘mycorrhizal’ LysM receptors seem to have lower sensitivity than their legume homologs [184]. Adding legume receptors, co-evolved for millions of years for specificity with their symbiont, to non-nodulating plants may help improve a new symbiosis but is unlikely to be necessary to trigger the CSP in response to the Nod factors of an engineered symbiont. Most legumes undergo “root hair infection” where a microcolony of rhizobia is enclosed by a curling root hair, and invagination of the host membrane forms an intracellular “infection thread” through which the bacteria move into the root cortex. However, root hair infection is dispensable for symbiotic nitrogen fixation, as demonstrated by nodulators with “crack entry” mechanisms such as peanut, and the *L. japonicus* mutants *root hairless* and *slippery root*, where the rhizobia enter the root via the crack formed by an emerged lateral root and form infection structures directly in the cortex [185]. The enclosure of bacteria in a host membrane (called the symbiosome) is likely an essential step for the efficiency of the symbiosis. However, this basal ‘infection module’, the group of genes that permit intracellular infection by microsymbionts, is conserved in all plants able to associate with arbuscular mycorrhizal fungi (Table 3). The genes that form this module are not yet fully characterized, but their conservation in nodulation and arbuscular mycorrhization is demonstrated by the example of *VAPYRIN* and *VAMP721d*/e, which are essential to both symbioses, as they establish the secretory pathway used to build the symbiosome during nodulation, and peri-arbuscular membranes during mycorrhization [190]. Symbiosomes in legumes are endocytosed from the plasma membrane, but in other nodulating plants, such as *Parasponia andersonii*, the infection thread remains contiguous with the plasma membrane, as it does in the arbuscular mycorrhizal symbiosis. Rhizobia still differentiate into bacteroids and fix nitrogen at high efficiency in these fixation threads, supporting the concept of intermediate stages of evolution that an engineering project could take advantage of [191].

**Table 3:** The common symbiosis pathway (CSP) controls the establishment of rhizobia–legume associations and the arbuscular mycorrhizal symbiosis

The common symbiosis pathway (CSP) controls the establishment of rhizobia–legume associations and the arbuscular mycorrhizal symbiosis.	
Arbuscular mycorrhizal fungi (Mucoromycotina) produce diffusible Myc factors composed of short chitin oligomers as well as lipo-chitooligosaccharides similar to rhizobial Nod factors. These fungal signals are perceived by LysM RLKs similar to the Nod factor receptors [62, 186, 187]. The arbuscular mycorrhizal association appeared with the first land plants about 450 million years ago and is still found in more than 70% land plants, including most legumes and cereals [188]. In contrast, root nodule symbioses appeared much more recently, around 100 million years ago, and are restricted to plants of the “FaFaCuRo clade” [182, 189]. It seems likely that the nitrogen-fixing bacteria mimicked fungal signals and co-opted the ancient and widespread mycorrhizal pathway.	

A critical missing link for nodulation outside the FaFaCuRo clade is likely the activation of ‘nodulins’ by the calcium oscillations of the CSP [72, 192]. Thus, the main challenge of the ‘minimum change' approach would be to add or alter promoter elements in an essential set of conserved ‘nodulin’ genes to coordinate their expression in response to nuclear calcium spiking. Some of the genes that make up this essential set are currently known (for example, NFRs, LYK3, CCaMK, IPD3/CYCLOPS, CASTOR/POLLUX, NIN, NSP1, NSP2, LHK1), but others would have to be elucidated through further research. One potential issue with this strategy is that it is contingent on how plants in the FaFaCuRo clade differentiate between arbuscular mycorrhizal and rhizobial signaling (as the same calcium spiking appears to elicit different gene expression, suggesting the existence of unknown secondary pathways [189, 193, 194]), but this is still a genetic black box beyond the scope of current knowledge).

In contrast to infection, shaping the organogenesis module of nodulation also may need only a few significant changes in non-nodulating plants. The basal actinorhizal plants produce nodules with central vasculature that arise from the pericycle, differentiated from lateral roots only by the cessation of growth and hosting of symbiosomes [34]. Nodules within the legumes are more elaborate, probably recruiting further genes to aid the symbiosis (for example, leghemoglobin for oxygen protection) [106, 195]. However, while these changes likely improve efficiency, they are probably dispensable and may be replaceable by bacterial functions [117, 196, 197]. So, what is necessary to trigger organogenesis of a nodule rather than a lateral root?

NIN, NF-Y proteins, and other components that regulate lateral root initiation in response to nitrogen starvation must be repurposed, activating their expression in the tissue layer destined to give rise to the nodule, in response to bacterial signaling. In legumes, this is characterized by a coordinated buildup of cytokinin and auxin to drive cell dedifferentiation and activation of the cell cycle, so links between these transcription factors and hormone synthesis will need to be confirmed or added in non-nodulating plants [67, 75]. A key difference between legume nodules with peripheral vasculature and lateral roots with a central vasculature appears to be controlled by homeotic transcription factors of the *NOOT* family. In the nodules of legume *noot* mutants the vasculature alternates between a peripheral or central location along the length of the nodule, an apparent reversion to a lateral root or to an actinorhizal nodule identity [198, 199]. *NOOT* orthologs are present in non-legumes, but their function is unknown.

Attempts to demonstrate the feasibility of this evolution-guided approach would be best undertaken in a close relative of the FaFaCuRo clade, to maximize the protein similarity of conserved ‘nodulins’. Of these relatives, poplar (*Populus* sp.) is an attractive model for the expansion of nodulation, given the ease of transformation and the phylogenetic proximity to the FaFaCuRo clade. The long-term objective of such approaches is, of course, to engineer root nodulation in cereals crops.

### Nitrogen-fixing associations outside the FaFaCuRo clade open new horizons

Engineering associative nitrogen fixation should, in theory, be more straightforward than engineering root nodules and intracellular infection or expressing the nitrogenase in plants. However, the expansion of symbiotic associative fixation faces a significant challenge due to the poor understanding of the genetic requirements that allow a host to associate with and benefit from diazotrophs. The benefit obtained by the host is likely governed by three factors: nitrogen uptake efficiency at low concentrations, defense responses, and the amount of carbon available to the diazotrophs. Blind manipulation of the latter two is likely to lead to problems with pathogens or competition from non-fixing rhizospheric microorganisms. Nitrogen uptake efficiency has been a breeding target, though it is often in a trade-off with the efficient uptake at high concentrations that intensity agriculture breeds for. Many crops benefit from some level of soil fixation (usually > 20 kg N ha^−1^ yr^−1^, but decreasing on nitrogen fertilization [123, 200, 201]), likely powered by photosynthates in root exudates. However, many more elaborate, and more efficient, nitrogen-fixing symbioses have been discovered in nature [12]. Of particular interest is the fixation on aerial roots of maize landraces from the Sierra Mixe [83]. These maize accessions produce aerial roots on many more nodes than conventional maize accessions. Upon rain, these roots secrete a sugar-rich mucilage, which houses diazotrophs that contribute 29–82% of the plant’s nitrogen [83]. Preliminary evidence suggests that tropical accessions of other cereals like sorghum may possess the same trait of abundant mucilage production by aerial roots [202]. Another example is Brazilian sugarcane, which obtains nitrogen from bacteria (most notably *Gluconacetobacter diazotrophicus*) housed within the stem, contributing up to 30% of the plant’s nitrogen [127]. The rate of biological nitrogen fixation is known to depend on the plant cultivar, and the phenotype seems dependent on the environment, but we are not aware of any exploration of the genetic basis of this trait [127, 203]. This type of associative nitrogen fixation provides an enormous well of untapped potential, and more efforts should be devoted to their study.

### Advantages and environmental concerns with nitrogen-fixing crops and microbes engineered for biological nitrogen fixation

Intensive agriculture leads to environmental degradation on a global scale. Microbial inoculants promise an alternative eco-friendly practice, reducing the amount of fertilizer usage. However, it is worth remembering that legumes themselves can lead to significant nitrogen leaching when crop residues are mineralized; thus, agronomic practices such as reduction of tillage and using cover crops must also be considered to solve these environmental issues [204]. Commercially available bioinoculants for non-legumes uses plant growth-promoting rhizobacteria, but the efficiency of these products in incorporating fixed nitrogen is still limited and variable depending on the environment (for an extensive review see [205]). *Azospirillum* is a versatile inoculant because it not only fixes nitrogen but also mineralizes nutrients from the soil and sequesters iron [206] (for a more comprehensive review see [207]).

On the other hand, endophytic bacteria, such as *Azoarcus* sp., *Herbaspirillum* sp., and *G. diazotrophicus*, appear promising candidates as they colonize the intercellular spaces, so fixed nitrogen is likely released directly to the plant without competition from the rhizosphere community [22, 120, 129, 145]. However, these endophytic bacteria display only a mild plant growth promotion effect. Thus, it is essential to improve the efficiency of the ammonium release from live microbes as opposed to relying on the release after cell death. It will also be necessary to understand better the microbial traits required for plant colonization, persistence, and competitiveness in the plant microbiota. Similarly, the impacts of plant growth-promoting rhizobacteria on endogenous microbial communities are understudied. The effect of these newcomers on preexisting microbial populations, and the useful ecosystem services which they provide, is unknown. One of the first studies to demonstrate the influence of the environment in the establishment of beneficial microbe–host interactions was conducted by Dr. Johanna Döbereiner, who showed in 1961 that the growth promoter of sugarcane, *Beijerinckia*, was dependent on rainfall [121]. Similarly, the rain on aerial roots of maize is required for secretion of mucilage [83]. More generally, the concept of a disease triangle in which host, microbe, and environment interact can be applied to beneficial microbes too. We believe that prospecting for better diazotrophs and better host plant genotypes combined with engineering approaches has the potential to deliver transformative agricultural tools and that different host–microbe combinations may be necessary for different environments.

#### Can we shortcut the bacteria and develop plants that fix nitrogen directly?

Engineering plants is generally more challenging than manipulating bacteria, primarily due to generation time and the bottleneck of plant transformation. However, a crop that fixes nitrogen without the need for microbes would have an agronomic impact without precedent. Current attempts to generate a nitrogen-fixing eukaryote have favored assembling the active nitrogenase inside chloroplasts or mitochondria. These organelles are the main sites of ATP synthesis, and so are most able to meet the high energetic requirements of the nitrogenase. López-Torrejón et al. showed that yeast mitochondria were anoxic enough to allow for the accumulation of active NifU and NifH and that, in the presence of NifM, NifH could incorporate endogenous mitochondrial Fe-S clusters [208]. Attempts to engineer transgenic yeast expressing nitrogenase have led to the identification of a minimal *nif* cassette of nine genes sufficient for nitrogen fixation. The stoichiometric ratios of these nine nitrogenase components are critical for the assembly of a functional holoenzyme [209]. Burén et al. showed that refactoring approaches could be used to recapitulate that in eukaryotes [210]. Assembly of large hetero-tetrameric complexes has proved challenging. The use of ‘giant gene’ constructs separated by peptides cleaved by the ribosome or proteases have been attempted, but the cleavage overhangs have been shown to impair both targeting and folding. Re-assemblies using giant genes have been demonstrated to fix nitrogen in *E. coli*, but functionality in a eukaryotic system is yet to be shown [211]. Allen et al. demonstrated that these lessons could be applied to plants, expressing 16 *nif* genes in the tobacco mitochondrial matrix [212]. For extensive reviews about strategies to transfer *nif* genes to eukaryotes refer to [209, 213].

If expressed in the chloroplast, the nitrogenase would be exposed to an ATP-rich environment and should not be exposed to high oxygen levels during the night [214]. Some cyanobacteria like *Synechococcus* perform photosynthesis during the day and fix nitrogen during the night, thus uncoupling photosynthesis and nitrogen fixation temporally [25]. The evolutionary relatedness of plant chloroplasts to cyanobacteria suggests that it may be possible to engineer such a “night shift” in plant chloroplasts. Ivleva et al. produced transplastomic tobacco plants expressing NifH/NifM, which was active in vitro under low oxygen conditions (10% O_2_) in the presence of the molybdenum-iron protein from *A. vinelandii* [215]. The current lack of evidence of nitrogenase function in eukaryotes, combined with the lack of a high-throughput plastid transformation procedure for monocots, means that the development of nitrogen-fixing cereals is still a long-term prospect.

### Advantages of and concerns with nitrogen-fixing crops

Developing plants that could fix and assimilate nitrogen without the help of microbial partners would alleviate the adverse effects of nitrogen fertilizers on the environment and benefit developing countries by facilitating higher yield in low input systems. Despite the genetic challenge, a plant capable of directly fixing nitrogen will be more robust than symbiotic nitrogen fixation, as it would decrease nitrogen loss to other organisms. Ammonium produced by nitrogenase could likely be coupled to plant metabolism in the plastid or mitochondria, further increasing efficiency [16, 209]. However, this approach will probably need much careful refinement, because if nitrogenase activity is not coupled to substrate delivery, the nitrogenase could divert large proportions of cellular resources to the futile evolution of hydrogen, imposing a significant yield drag on the plant [216]. Another considerable advantage of self-fixing plants would be the freedom from the partner requirement of symbiotic nitrogen fixation, as germline transmission would provide for more straightforward distribution and require less infrastructure from the farmer, compared to a symbiotic nitrogen fixation approach which would require inoculation. Concerning food security, transgenic plants are regulated and cultivated in many countries, and so far no soil, environment, or health issues have been correlated to it. It is necessary that the current regulation is revisited to avoid unnecessary fears preventing society from benefiting from this technology, which has the potential to make food production more environmentally sustainable and help feed the increasing world population.

#### Conclusions and perspectives

The Food and Agriculture Organization of the United Nations estimates that the Earth will have two billion more people to feed in 2050 [217]. Given that half of the world population is currently sustained through synthetic fertilizers, it would not be reasonable to claim that biological nitrogen fixation will replace the Haber-Bosch process entirely. But, as indicated earlier, the extreme dependence of the global food supply on synthetic fertilizers is not sustainable. Now is the time for a “Symbiotic Revolution” to combine food production and sustainable soil health. So, are we there yet?

**Fig. 2:**
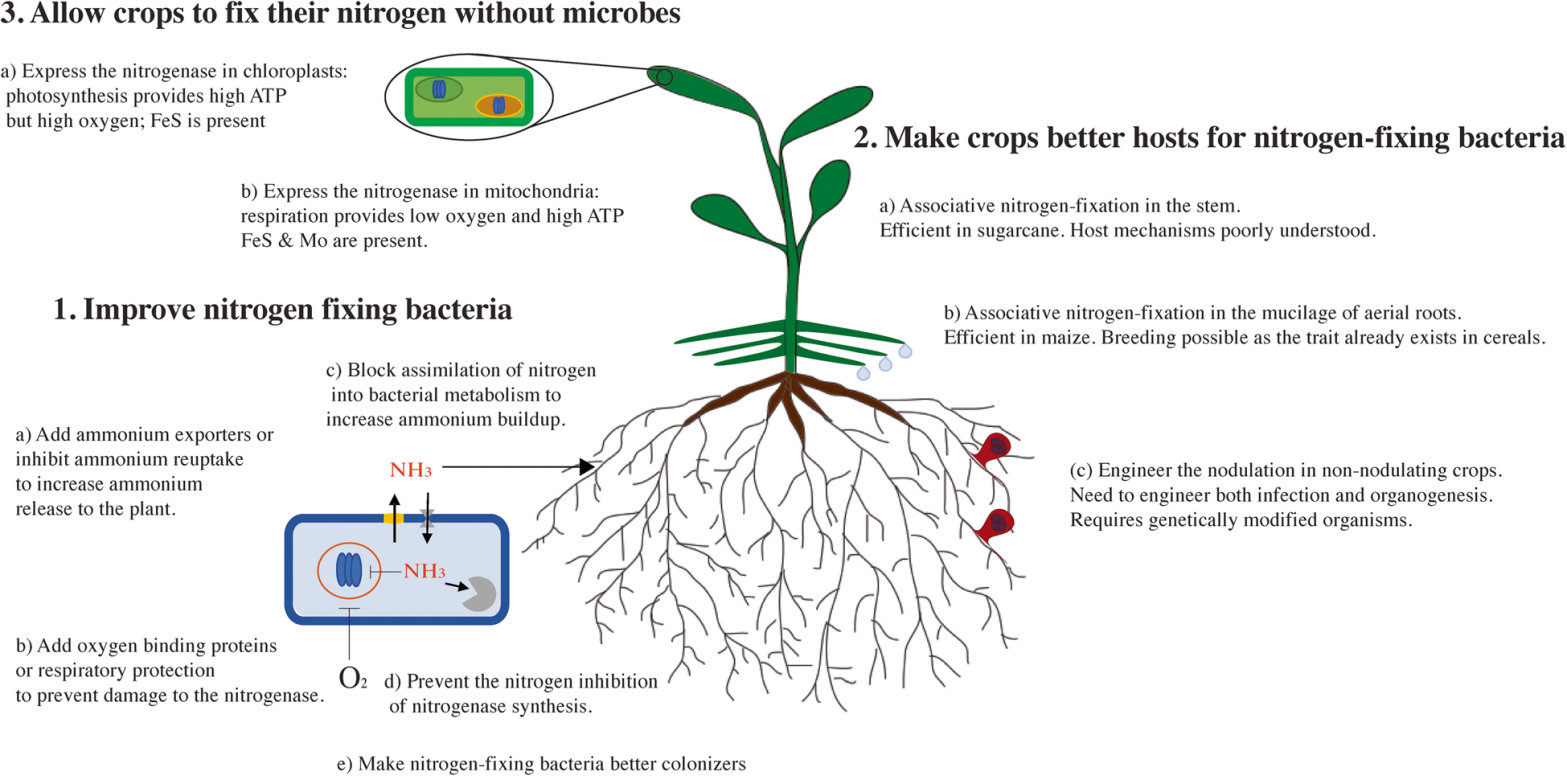
Main approaches to engineer or improve biological nitrogen fixation in cereals. *1* Improving nitrogen-fixing bacteria: (*a*) [153], (*b*) [111, 113], (*c*) [154], (*d*) [152], (*e*) [110, 144, 171]. *2* Making crops better hosts for nitrogen-fixing bacteria: (*a*) [127], (*b*) [83], (*c*) [56]. *3* Allowing crops to fix their nitrogen without microbes [209]

Many avenues to improve biological nitrogen fixation in non-leguminous crops have been described in this review (Fig. 2). Some of them could bring solutions in the next decade, and some will probably bear fruit in the longer term [83, 128, 208, 211, 212]. Some of the natural systems we presented can provide significant amounts of biologically fixed nitrogen. Microbial and plant natural diversity is a resource and a source of knowledge that should be explored more, and that could deliver practical solutions in a relatively short time. We take as an example the Sierra Mixe maize, where an unexplored system is capable of sustaining most of the nitrogen requirement for the crop over several months and at a critical period of the growing season [83]. Such unexpected discoveries reinforce the need to preserve natural diversity in our crops and their wild relatives. These Sierra Mixe landraces cannot be used directly in most cropping systems or environments due to their size and their long growing season. Breeding the trait in more conventional accessions of maize is necessary, but this process will take time. Once this trait is introduced into more conventional varieties, agronomic questions such as the amount of fertilizer saved by the trait, the effect of soil nitrogen, or the yield cost of the trait will need to be addressed. This will require efforts and funding, but it seems achievable to use such natural traits in the next decades. Approaches aiming at engineering root nodules in cereals are more complex and will likely take more time. Procedures to engineer crops able to fix their nitrogen without the bacteria seem even longer term. Nevertheless, as discussed previously, these long-term approaches are promising and likely to succeed.

A critical justification for pursuing the range of approaches described in this review is the significant effect that the environment has on many of these biological systems. Taking the example of the Sierra Mixe maize again, mucilage production by the maize aerial roots is dependent on rain [83]. While this trait seems to be directly usable in many regions of the world, it will be more challenging to adapt it to arid environments. If we look at the worldwide distribution of nodulating legumes, engineered root nodules may be efficient in a broader range of situations, but legume nodulation itself is affected by environmental factors such as soil nitrogen or flooding [218–220]. The environmental dependence of plants fixing their own nitrogen is, at this point, entirely speculative.

Lastly, as indicated earlier, the process of nitrogen fixation, whether biological or industrial, requires significant amounts of energy. In all the approaches to improve nitrogen fixation discussed in this review, the energy for nitrogen fixation comes from plant photosynthesis and will have a cost on plant carbon. Despite the tight autoregulation of nodulation, legumes dedicate 10–20% of their carbon to nodules [117]. This does not necessarily decrease yield, as carbon cost is offset by increased photosynthetic capacity due to the nitrogen gained from biological nitrogen fixation. However, symbiotic nitrogen fixation will not be energetically competitive compared to nitrogen fertilization, and growers in developed countries are not ready to suffer any yield loss while fertilization remains cheap. Estimating the potential yield penalty for different strategies will be necessary. Environmental policies may provide more incentives for a reduction of synthetic fertilizers in the future [221]. In developing countries, any nitrogen input will be valuable for poor farmers where nitrogen is the most important factor limiting their production [222]. International projects such as Realizing Increased Photosynthetic Efficiency (RIPE) are currently working on improving photosynthetic efficiency, which could offset the yield penalty of relying on biological nitrogen fixation [223, 224].

Improving nitrogen fixation in non-leguminous crops has been a dream of the agronomic community for more than a century. The global challenges that our world is facing make the realization of this dream urgent. Fortunately, natural diversity holds solutions that the scientific community overlooked possibly because of the intense focus on legume nodules. Technological developments such as the advent of next-generation sequencing, gene editing, and synthetic biology allow the dissection and manipulation of plants and microbes at an unprecedented scale. We are confident that combining the prospecting of plant and bacterial natural diversity with genetic engineering will deliver solutions in the short and long terms and will help to feed the world in a more sustainable manner.

## Data Availability

Not applicable.
